# Personomics: The Missing Link in the Evolution from Precision Medicine to Personalized Medicine

**DOI:** 10.3390/jpm7040011

**Published:** 2017-10-16

**Authors:** Roy C. Ziegelstein

**Affiliations:** Johns Hopkins University School of Medicine, Baltimore, MD 21205, USA; rziegel2@jhmi.edu; Tel.: +1 410-955-8401

**Keywords:** personomics, precision medicine, health care, personalized medicine

## Abstract

Clinical practice guidelines have been developed for many common conditions based on data from randomized controlled trials. When medicine is informed solely by clinical practice guidelines, however, the patient is not treated as an individual, but rather as member of a group. Precision medicine, as defined herein, characterizes unique biological characteristics of the individual or of specimens obtained from an individual to tailor diagnostics and therapeutics to a specific patient. These unique biological characteristics are defined by the tools of precision medicine: genomics, proteomics, metabolomics, epigenomics, pharmacogenomics, and other “-omics.” Personalized medicine, as defined herein, uses additional information about the individual derived from knowing the patient as a person. These unique personal characteristics are defined by the tools of personalized medicine—personomics—which take into account an individual’s personality, preferences, values, goals, health beliefs, social support network, financial resources, and unique life circumstances that affect how and when a given health condition will manifest in that person and how that condition will respond to treatment. In this paradigm, precision medicine may be considered a necessary step in the evolution of medical care to personalized medicine, with personomics as the missing link.

There is much discussion today about “precision medicine” and “personalized medicine.” Although these terms were not in common use a century ago, physicians in 1917 might well have described their practice using these terms. Physicians generally knew their patients well as people. Physicians and patients often shared the same local community, schools, stores, house of worship, current events, and gossip. Although medicine may not have been well-grounded in science, medical care was personally tailored to the individual.

Although physicians a century ago may have practiced a type of personalized medicine, it would be difficult to say that personalized medicine is the norm today, however defined. The last several decades in medicine have been marked by the generation of masses of data from randomized controlled trials (RCTs) that have led to the development of a new field of evidence-based medicine (EBM). EBM has, in turn, led to the generation of clinical practice guidelines for many, if not most, common conditions, not only based on RCTs, but on meta-analyses of those trials. In fact, in many countries, incentives are provided to physicians whose practice is consistent with these guidelines, with penalties to those whose practice is not. When medicine is informed strictly by clinical practice guidelines, the patient is not treated as an individual, but rather a member of a group. Individual variation is certainly not the driver of medical practice; instead, care is based on groups of individuals with similar characteristics who have a similar health condition at a similar stage of progression. In this paradigm, treatment recommendations are often guided by predictions about prognosis and by assessments of risks and benefits that are based on groups of patients felt to be similar to the patient being treated. Indeed, the process of prognostication is—like the creation of an actuarial life table—by definition, treating each individual as if he or she is not unique. It is based on the notion that predictions about an individual can be made based on the prior experience of a group of people felt to have similar characteristics in what is felt to be a similar situation.

As we consider the practice of medicine today and the ideal practice of medicine in the future, we should begin by defining the terms precision medicine and personalized medicine. This commentary proposes an operational definition of these terms and the use of a recently coined term—personomics [1]—to help differentiate the two terms. Herein, precision medicine is defined as the application of recent advances in medical science to characterize individuals based on the unique biological characteristics of the individual or of specimens obtained from that individual. Precision medicine uses information derived from genomics, proteomics, metabolomics, epigenomics, pharmacogenomics, and other “-omics” to derive more precisely tailored diagnostics and therapeutics and thereby improve human health [2]. For the data from precision medicine to be used optimally to improve human health, systems biology will need to collect and integrate data “…from the molecular level, through cells, tissues and organisms, to the population level” [3]. Systems biology will need to be applied to human disease in what has been termed systems medicine, [4] using high throughput technologies to produce and integrate enormous data sets that lead to an improved understanding of human biology. It has been suggested that as digital technology allows these data to be communicated more readily to activated patients and consumers, the possibility of a new healthcare system may be realized that is predictive, preventive, personalized, and participatory (P4) [3].

Personalized medicine adds to that information derived from knowing the patient’s unique psychosocial situation, taking into consideration the individual’s personal preferences and health beliefs, as well as the individual’s values and goals. As much as genomics, proteomics, metabolomics, epigenomics, pharmacogenomics, and other “-omics” are the tools of precision medicine, personomics [1] can be used to describe the tools of personalized medicine. Personomics recognizes that individuals are not only distinguished by their biological variability, but also by their personalities, health beliefs, social support networks, financial resources, and other unique life circumstances that have important effects on how and when a given health condition will manifest in that individual and how it will respond to treatment [1]. The concept of personomics emphasizes that these components of individuality are as critical to patient care as any of the more traditional “-omics” noted previously. Just as these other “-omics” allow for an improved understanding of the pathogenesis and treatment of disease in precision medicine, personomics facilitates the delivery of personalized medicine by enabling physicians to develop a better understanding and appreciation of the patient’s environment and life circumstances. Personalized medicine calls for a deeper understanding of each individual’s health behaviors, since behavioral habits such as cigarette smoking, unhealthy diet, and lack of exercise are responsible for many important health conditions that result in premature death [5]. Personalized medicine also means that physicians must understand each patient’s financial circumstances, since what diagnostic tests and treatments are appropriate for a patient is based not only on the individual’s genomics, proteomics, and metabolomics, but also on what tests and treatments that person can afford. Precision medicine may be considered a necessary step in the evolution of medical care to personalized medicine, with personomics as the missing link. The evolution from precision medicine to personalized medicine is the evolution from “health care” to “health caring.” As Chochinov noted, “Where health care asks about a problem, health caring asks, ‘What do I need to know about you as a person to give you the best care possible?” [6].

To understand this evolution—over the last 25 years—of medical care informed by clinical practice guidelines to a precision medicine approach, and then to think about how personalized medicine might be practiced, it may be useful to consider the treatment of a 72-year-old woman with node-positive, estrogen receptor (ER)-positive breast cancer diagnosed on a routine mammogram. In 1992, this patient’s care would have been informed by an important publication from the Early Breast Cancer Trialists’ Collaborative Group on the systemic treatment of early breast cancer by hormonal, cytotoxic, or immune therapy [7]. In that report of 133 RCTs involving 75,000 women, systemic chemotherapy was shown to significantly reduce the risk of recurrence among women with early stage breast cancer. However, very little information was presented from women 70 years of age and older, since most RCTs at the time excluded this age group. The effect of systemic chemotherapy in this age group was therefore statistically uncertain. Indeed, the report itself notes that the reviewed trials “… provide virtually no information about women aged over 70” [7]. A physician treating this 72-year-old woman with node-positive, estrogen receptor (ER)-positive breast cancer in 1992 might well have recommended forgoing systemic chemotherapy based on the paucity of data in this age group, even if the patient in question were otherwise healthy.

The situation for this woman would be different today, since precision medicine allows us to base her treatment recommendations on the classification of her tumor into specific molecular subtypes identified from the analysis of genomic alterations in tumor tissue [8]. Predicted survival of women with breast cancer is no longer based solely on the results of RCTs stratified by age and menopausal status, but also based on breast cancer genomic variability and on the status of certain breast cancer receptors, namely the ER, the progesterone receptor (PR), and the human epidermal growth factor receptor 2 (HER2). ER/PR/HER2 status is known to affect patient survival (with so-called triple negative patients having the worst survival) and helps physicians select appropriate treatment regimens [9]. A host of targeted therapeutic options would now be available to be used based on the genomic profiling of this woman’s tumor [10]. The physician’s recommendation about systemic chemotherapy would be informed by the 21-gene recurrence score assay that may be used to stratify the risk of recurrence of this woman’s type of early-stage, ER-positive breast cancer [11]. This is the essence of precision medicine.

Precision medicine would also recognize if this woman’s tumor was among the 15–20% of breast cancers that overexpress HER2, a finding that is associated with aggressive tumor behavior and reduced survival [12,13]. If this woman’s tumor overexpressed HER2, today’s oncologist would be able to employ a treatment such as trastuzumab, a humanized monoclonal antibody that blocks HER2 activity [14], in addition to systemic chemotherapy. It has been shown that women with HER2-positive breast cancer who received trastuzumab have a 44% lower risk of death compared even to women with HER2-negative breast cancer [15]. In another study, the addition of trastuzumab to chemotherapy in the treatment of women with HER2-positive metastatic breast cancer decreased the risk of death at 1 year from 33% to 22% (*p* = 0.008) and increased median survival from 20.3 to 25.1. months (*p* = 0.01) [14]. Today’s oncologist would also be able to treat this woman with lapatinib, a small molecule that reversibly inhibits HER1 and HER2, and has been used in women with HER2-positive advanced breast cancer that has progressed after treatment with conventional chemotherapy and trastuzumab [16]. A recent meta-analysis of randomized controlled trials compared the effects of trastuzumab, lapatinib, alone and in combination, in HER2-positive breast cancer. The meta-analysis included 2350 patients, 837 who received lapatinib, 913 who received trastuzumab, and 555 who received the combination. It showed that while trastuzumab is first-line therapy in women with HER2-positive breast cancer, combination therapy increases the pathological complete response rate compared to trastuzumab alone [17].

Despite these advances, there are limitations to precision medicine, even in the field of oncology. Tannock and Hickman recently reviewed trials of cancer therapy guided by genetic sequencing and noted that “The outcomes of these investigations are discouraging” [18]. While it is possible that these limitations will improve as the science of molecular targeted treatments matures, these authors suggested that benefits will be less than expected because of inherent limitations of targeted therapies and because of intratumor heterogeneity. They noted that targeted therapies only partially inhibit critical signaling pathways and that related signaling pathways are also present in normal cells [18]. Given this, currently existing treatments often exhibit significant dose-limiting side effects. Since cancer cells typically develop resistance to agents that target a single signaling pathway, combinations of targeted therapies that inhibit different pathways are often needed. However, side effects often limit a patient’s ability to tolerate effective doses of these combination targeted treatments.

How would this woman be treated using a personalized medicine approach? Using the tools of personomics, the physician would get to know this patient as an individual. The physician would certainly consider the patient’s age and general health status, and the physician would most definitely inform treatment decisions based on the molecular subtype of this woman’s tumor. In addition, though, the physician would ask and learn about this woman’s values and goals. The physician would ask and learn about the support this woman would likely receive (or not) from family and friends. This support would be needed regardless of the type of treatment used, and it may be more likely to be needed in the short term if cytotoxic chemotherapy is recommended or if molecular targeted treatments are used that may result in dose-limiting side effects. The physician would ask and learn about this woman’s health beliefs, and in particular whether she favors doing whatever possible to prolong life or whether quality of life is more important to her. The physician would ask and learn who is most important in the patient’s life and the extent to which she depends on these individuals to make critical health care and life decisions. The physician would ask about this woman’s financial circumstances and health insurance coverage and consider her ability to afford the type of medications that might be needed to treat this tumor type, since some of these medications—in particular many of the newer targeted therapies—may be very expensive and might not be covered by some forms of health insurance. The physician who practices personalized medicine would base a recommendation about systemic chemotherapy and other targeted therapies on this woman’s age, general health, recurrence score testing, and all the information gleaned from putting personomics into practice. In summary, the evolution from medical care based on clinical practice guidelines to precision medicine to personalized medicine is, in many ways, an evolution from lumpers to splitters. Clinical practice guidelines lump patients together based on certain shared characteristics. As Sacristan and Dilla note, “… guidelines are based on the results of large RCTs and meta-analyses containing information that, in theory, is applicable to ‘average patients’. However, doctors do not treat average patients; thus, this ‘generalizable knowledge’ that may be useful to standardize medical practice is not the most appropriate to treat individual patients” [19]. Precision medicine, by contrast, splits patients into groups defined by biological characteristics and allows for the delivery of more individualized medical care. In the case of cancer treatment, the biological characteristics are based largely on analysis of tumor tissue. As NIH Director Francis Collins noted, “If I had cancer diagnosed today, I would want that cancer to be completely analyzed. I would want to know ‘what are the drivers in my tumor that are making it do those bad things,’ and then I’d want to compare that to a menu of targeted drugs…” [20]. Personalized medicine splits patients further, recognizing that knowledge of the patient as a person—personomics—is necessary to deliver truly individualized care to every patient based on the unique circumstances of the individual.
